# Microscopic colitis found together with celiac disease in a female population is associated with one episode of lymphocytic colitis

**DOI:** 10.1186/s12876-024-03158-2

**Published:** 2024-02-12

**Authors:** Bodil Roth, Bodil Ohlsson

**Affiliations:** Department of Internal Medicine, Lund University, Skåne University Hospital, Jan Waldenströms Street 15, Floor 5, 205 02 Malmö, Sweden

**Keywords:** Microscopic colitis, Celiac disease, Smoking habits, Corticosteroids, Collagenous colitis, Lymphocytic colitis

## Abstract

**Background:**

Microscopic colitis (MC) is considered a chronic disease associated with autoimmune disease, smoking, and drugs. The aim was to examine the association between MC and celiac disease, adjusted for smoking, considering subtypes and clinical course of the disease in a retrospectively collected female cohort.

**Methods:**

Women (*n* = 240), ≤ 73 years, diagnosed as MC in medical records or pathological registers were invited. One hundred and fifty-eight women accepted to be included. Participants completed a study questionnaire about sociodemographic factors, lifestyle habits, and medical history; the Rome III questionnaire; and the visual analog scale for irritable bowel syndrome (VAS-IBS). Participants were categorized into collagenous colitis (CC) (*n* = 92) and lymphocytic colitis (LC) (*n* = 66) or MC with one episode of the disease (*n* = 70) and refractory MC (*n* = 88). Presence of IBS-like symptoms were noted. Blood samples were collected and analyzed for anti-transglutaminase antibodies. Differences between groups were calculated and logistic regression was adjusted for smoking habits.

**Results:**

MC and celiac disease debuted simultaneously in half of the cases. Celiac disease was most prevalent in LC (12.1% vs. 3.3%; *p* = 0.05) and MC with one episode (12.9% vs. 2.3%; *p* = 0.01). Anti-transglutaminase antibodies were found in one patient with one episode of MC. Corticosteroid use was most often found in CC (37.0% vs. 21.2%; *p* = 0.037) and refractory MC (38.6% vs. 20.0%; *p* = 0.015). Past smokers were most prevalent in patients with one episode of MC (54.3 vs. 29.5%; *p* = 0.007). Current smoking was the smoking habit with highest prevalence of IBS-like symptoms. When adjusted for smoking habits, celiac disease was associated with LC (OR: 4.222; 95% CI: 1.020–17.469; *p* = 0.047) and tended to be inversely associated with refractory MC (OR: 0.210; 95% CI: 0.042–1.506; *p* = 0.058).

**Conclusion:**

Celiac disease is most common in patients with one episode of LC. The question remains whether LC in combination with celiac disease should be classified as celiac disease or two different entities.

## Background

Microscopic colitis (MC) is an inflammatory disease of the colonic mucosa that predominantly affects elder women, with an average age of 65 years at diagnosis, and a three- to fourfold increased prevalence in women compared with men [1]. The most common clinical presentation is chronic, non-bloody diarrhea with normal or close to normal endoscopic findings [1]. MC can be divided into collagenous colitis (CC) and lymphocytic colitis (LC) [2, 3]. The histological criteria for CC are a thickened subepithelial collagen layer (> 10 µm) in the extracellular matrix of the mucosa, epithelial damage, and presence of an inflammatory infiltrate in the lamina propria [4]. The criteria for LC are > 20 intra-epithelial lymphocytes/100 enterocytes [4].

The pathophysiology to MC is unknown. The most well-documented etiological finding of MC is that present smoking, and to a lesser extent also past smoking, show a clear association with MC [1, 3, 5], and may be one explanation to the higher incidence of MC in Denmark compared with Sweden [6]. MC has in several studies been found to be associated with celiac disease, autoimmune thyroid disease, Sjögren’s syndrome, diabetes mellitus, and several conditions of the skin and joints [1]. The concomitant presence of celiac disease was 4.4% in MC [7] and 12.9% in CC [5]. The association of MC with celiac disease and autoimmunity was stronger at younger age in nationwide Swedish and Danish studies [8, 9]. Medication may be another etiology to MC [1, 10].

Familial occurrence of CC has been described [11]. This rendered examination with genome-wide association studies (GWAS), which revealed associations between CC and human leucocyte antigen (HLA) variants, also associated with several autoimmune diseases [12]. This may explain the observed comorbidity with autoimmunity in patients with CC and their first-degree relatives, and that CC truly represents a disease of immune-mediated nature [12]. These findings were different from LC, which did not show any significant HLA associations in GWAS [13].

Pathologists describe that a microscopic inflammation can be found throughout the gastrointestinal mucosa in celiac disease, and therefore, the connection between celiac disease and MC may in some cases represent one entity instead of two different entities [14]. Since the colon mucosa lack villi, the colonic reaction to gluten intolerance could hypothetically instead lead to infiltration of lymphocytes and other inflammatory cells into the lamina propria. This is supported by highest association between celiac disease and LC [9] and clinical improvement and healing of the LC after introduction of a gluten-free diet in 4 of 9 patients [15]. However, both colonic mucosal alterations and intestinal villus atrophy may sometimes be resistant to dietary changes [14, 15].

Since the hypothesis has been raised that MC may be the colonic reaction to gluten in celiac disease [9, 14, 15], we wanted to retrospectively examine the association between the debut of celiac disease and MC in a female cohort. The aim of the present extended study was therefore to examine the association between MC and celiac disease, adjusting for smoking habits, when considering both the subtype of MC, gastrointestinal symptoms, and the clinical course of the disease.

## Methods

### Patients and study design

Women treated for MC at any outpatient clinic of the Departments of Gastroenterology in Skåne, Sweden, between 2002 and 2010, were retrospectively identified by search for the ICD-10 classification of the two forms CC and LC (K52.8) in medical records, as well as in the local register at the Department of Pathology, Skåne University Hospital, Malmö. About one-third of identified patients were excluded due to age over 73 years, since elder patients had many other concomitant diseases and drug therapies. Of the patients recognized, 240 patients were ≤ 73 years old and had their diagnosis verified by colonic biopsy according to the criteria: 1) a thickened subepithelial collagen layer (> 10 µm); 2) epithelial damage; and 3) an inflammatory infiltrate in the lamina propria [2], or > 20 intra-epithelial lymphocytes/100 enterocytes [3].

Between March and June 2011, invitations and information were sent to all recognized patients. Questionnaires regarding sociodemographic factors, lifestyle habits, and medical data (study questionnaire), the Rome III questionnaire, and the visual analog scale for irritable bowel syndrome (VAS-IBS) regarding gastrointestinal symptoms were dispatched by post to assess the status at the time of inclusion. A reminding letter was sent a month after the first invitation letter to those who had not answered to the first letter.

Of the 240 invited patients, 159 patients accepted to participate in the study. Later, one was excluded due to the diagnosis of inflammatory bowel disease (IBD), rendering 158 patients finally included (62.9 (range: 27.0–73.1; interquartile ranges (IQR): 58.0–67.3) years). Of these, 133 visited the clinic and delivered blood samples, which were centrifugated and kept in -20^◦^ C until later analysis of tTG IgG antibodies in serum. At the same time, the completed questionnaires were received. Those who did not visit the clinic, returned the questionnaires by post. Medical records were scrutinized, and age, gastrointestinal symptoms, examinations, diagnoses, and treatments were recorded. Presence of celiac disease was registered, when the diagnosis was set in the medical record after examination of anti-human tissue transglutaminase (tTG) IgG in serum and histopathological verification of infiltrative, hyperplastic, and atrophic lesions in the small intestine according to modified Marsh criteria [16, 17].

### Questionnaires

#### Study questionnaire

A study questionnaire about marital status, education, employment, snuff and smoking habits, alcohol consumption, medical conditions they ever had been treated for, and medication was completed by all participants. The participant had to reply to one specific question for each common disease whether they ever had been treated for it or not.

#### Rome III criteria

The patients completed a shortened version of the Rome III questionnaire, including only IBS symptoms [18]. Patients who fulfilled the criteria for Rome III were classified as suffering from IBS-like symptoms. Since their diagnosis was MC, it cannot be called IBS [19].

#### Visual analog scale for irritable bowel syndrome

The VAS-IBS is a short, psychometrical test developed to assess gastrointestinal symptoms and psychological well-being during the past 2 weeks [20]. The questionnaire includes one VAS scale for each of the symptoms abdominal pain, diarrhea, constipation, bloating and flatulence, vomiting and nausea, perception of psychological well-being, and the intestinal symptoms’ influence on daily life, graded from 0 to100 mm, with 100 mm representing the worst symptoms. The scale is inverted from the original version [20]. Reference values for healthy women are available [21].

### Categorization

Participants were divided into the subtypes CC and LC based on the histopathology. At the time point of inclusion, many of the patients explained that they felt healthy and did not experience that they suffered from any gastrointestinal disease. Therefore, the patients were further classified as suffering from refractory MC when there was a history of at least two episodes of watery diarrhea; and/or dependence on long-term treatment of corticosteroids to maintain remission; and/or two pathological intestinal mucosa biopsies, in line with criteria suggested for diagnosing IBD [22, 23]. The other MC group included patients who had had only one episode of severe diarrhea (diarrhea that rendered examination by colonoscopy) with a pathological biopsy and then normal biopsies on later examinations in combination with a clinical remission or IBS-like symptoms. The patients were also classified depending on whether concomitant IBS-like symptoms were present or not.

Education was divided into university degree or not since most subjects in the society has completed secondary school. Occupation status was divided into employed, retired, or others, which included studying, sick-leave, and un-employment. Married or living together was collected in one group. Smoking was divided into never smoking, past smoking, and present smoking. Use of snuff or nicotine chewing gums were recorded. The number of days drinking alcohol during a month was given, as well as the number of standard glasses consumed on each occasion of alcohol drinking.

### Immunological analyses

At inclusion, all 133 patients who had delivered blood samples, 76 patients with CC and 55 with LC, 58 patients with one episode of MC and 73 with refractory MC, were screened for anti-tTG by enzyme-linked immunosorbent assay (ELISA) according to clinical routines at the Department of Clinical Chemistry [24]. A second screening was performed in 37 patients with refractory MC. Analysis of a combination of IgG anti-deamidated gliadin peptide (DGP) and IgA anti-tTG has shown high specificity and sensitivity in screening [25]. Thus, the ImmuLisa™ Celiac tTG IgG Ab Enhanced ELISA (Immco Diagnostics Inc., Buffalo, NY, USA) solid-phase immunoassay was used according to the manufacturer´s instructions for qualitative and semi-quantitative detection of anti-tTG antibodies in serum [26]. Results are expressed as ELISA units/milliliter and reported as positive or negative (qualitative determination), where < 20 EU/ml means negative, 20–25 EU/ml mean borderline, and > 25 EU/ml means positive values. According to the manufacturer (Immco Diagnostics Inc), six of 112 healthy men and women (5.4%) are positive in this test. Intra-assay coefficient of variation (CV) was 5.3% and inter-assay CV was 9.4%.

### Statistical analyses

Statistical analyses were performed using software SPSS^©^, version 28, for Windows (IBM, New York, USA). Since the values were not normally distributed, comparisons between groups were performed by Mann–Whitney U test or Kruskal–Wallis test when comparing more than two groups. Fisher’s exact test was used for dichotomous variables. Values are given as median and IQR or number and percentages. Logistic regression was used with CC/LC or one episode of/refractory MC as dependent variables and smoking and celiac disease as independent variables, to determine odds ratio (OR) and 95% confidence interval (CI). *P* ≤ 0.05 was considered statistically significant.

## Results

### Group characteristics

Of the 158 patients included, 92 participants were diagnosed as CC and 66 participants were diagnosed as LC according to the medical records. The disease duration at study inclusion ranged between 1–50 years, mean 10.9 ± 10.1 years, median 8.0 (4.0–14.0) years. More patients in the CC group were married/living together than in the LC group (Table 1). Celiac disease was more common in the LC group (12.1%) compared with the CC group (3.3%) (*p* = 0.050). The use of corticosteroids was most common in the CC group (37.0% vs. 21.2%; *p* = 0.037). The patients were often treated with proton pump inhibitors (25.9%), antidepressant drugs of the type of selective serotonin reuptake inhibitors (SSRI) (19.0%), statins (17.7%), and thyroid hormones (16.5%), with no differences between the groups except SSRI which was most prevalently used in the LC group (Table 2). There were no differences in symptoms or current or previous comorbidities according to the histopathological feature (Table 2).

**Table 1:** Basal characteristics depending on histopathological classification

	**Collagenous colitis** ***N* = 92**	**Lymphocytic colitis** ***N* = 66**	***P*-value**
**Age** (year)	63.3 (59.0–68.3)	62.6 (53.8–66.8)	0.178
**BMI** (kg/m^2^) *Missing value*	24.7 (21.8–29.2) 39	24.9 (23.0–27.4)	0.941
**Refractory MC**	56 (60.9)	32 (48.5)	0.145
**IBS**	49 (53.3)	38 (57.6)	0.629
**Disease duration** (years) *Missing value*	8.0 (3.0–13.5) 7	8.0 (4.0–16.0) 8	0.480
**University education** *Missing value*	28 (30.4)	26 (39.4)	0.495
**Occupation**			0.778
Employed	42 (45.7)	28 (42.2)	
Retired	44 (47.8)	35 (53.0)	
Others	6 (6.5)	3 (4.5)	
**Married/living together** *Missing value*	58 (63.0)	31 (47.0)	0.050
**Smoking**			0.788
Never smoking	23 (25.0)	18 (27.3)	
Past smoking	36 (39.1)	28 (42.4)	
Present smoking	33 (35.9)	20 (30.3)	
**Snuff users** *Missing value*	2 (2.2) 3	2 (3.0) 3	1.000
**Nicotine chewing gum** *Missing value*	11 (12.0) 2	3 (4.5) 1	0.155
**The number of days drinking/month** *Missing value*	6 (2–10) 25	4 (2–10) 16	0.457
**Number of standard glasses each day of drinking**			0.156
Never drinking	5 (5.4)	10 (15.1)	
1	13 (14.1)	4 (6.1)	
2	29 (31.5)	21 (31.8)	
3	7 (7.6)	5 (7.6)	
4	2 (2.2)	3 (4.5)	
≥ 5		2 (3.0)	
Missing value	36	21	

*BMI* body mass index, *IBS* irritable bowel syndrome, *MC* microscopic colitis. Values are given as number and percentage and median and interquartile range. Mann–Whitney U test and Fisher´s exact test. *P* ≤ 0.05 was considered statistically significant

**Table 2:** Comorbidity and symptoms depending on histopathological classification

	**Collagenous colitis** ***N* = 92**	**Lymphocytic colitis** ***N* = 66**	***P*-value**
**Comorbidity**			
Hypertension *Missing value*	34 (37.0) 10	20 (30.3) 6	0.572
Rheumatoid arthritis *Missing value*	18 (19.6) 13	15 (22.7) 9	
Asthma and/or bronchitis *Missing value*	16 (17.4) 11	9 (13.6) 12	0.632
Diabetes *Missing value*	6 (6.5) 13	5 (7.6) 11	0.694
Celiac disease *Missing value*	3 (3.3) 17	8 (12.1) 13	0.050
Gastric ulcer *Missing value*	12 (13.0) 14	11 (16.7) 8	0.749
Thyroid disease *Missing value*	16 (17.4) 15	12 (18.2) 10	1.000
Cancer *Missing value*	10 (10.9) 15	3 (4.5) 11	0.770
**Drug treatment**			
Corticosteroids	34 (37.0)	14 (21.2)	0.037
Proton pump inhibitors	23 (25.0)	18 (27.3)	0.854
Selective serotonin reuptake inhibitors	11 (12.0)	19 (28.8)	0.013
Statins	17 (18.5)	11 (16.7)	0.835
Thyroid hormones	15 (16.3)	11 (16.7)	1.000
**Gastrointestinal symptoms**			
Abdominal pain 5 (1–15)	35 (12–53)	41 (12–57)	0.196
Diarrhea 3 (0–10)	40 (14–77)	50 (26–73)	0.289
Constipation 9 (1–22)	6 (2–18)	9 (4–28)	0.085
Bloating and flatulence 14 (1–29)	55 (20–77)	55 (25–71)	0.805
Vomiting and nausea 2 (0–3)	7 (1–23)	13 (2–38)	0.130
Intestinal influence on daily life 2 (0–18)	45 (12–74)	42 (26–84)	0.151
Psychological well-being 4 (0–16)	26 (10–54)	24 (10–50)	0.751

Comorbidity reflects whether the patients ever have been treated for any of these diseases. Missing values mean that the participant has not answered the question whether they have had the disease or not in the questionnaire. Gastrointestinal symptoms were assessed by the Visual Analog Scale for Irritable Bowel Syndrome, 0–100 mm, where 0 represents no symptoms and 100 maximal symptoms [20]. Reference values for VAS-IBS from healthy women are shown within brackets [21]. Values are given as number and percentages or median and interquartile ranges. Mann–Whitney U test or Fisher´s exact test. *P* ≤ 0.05 was considered statistically significant

When considering the clinical course of the disease, 88 patients suffered from refractory MC and 70 from one episode of MC. Most patients in the group of one episode were former smokers (54.3%). Present smoking was the most prevalent smoking habit in refractory MC (39.8%) (Table 3). Celiac disease was present in 12.9% of the patients with one episode of MC, whereas only 2.3% in the refractory group suffered from celiac disease (*p* = 0.010). The distribution of other previous or concomitant diseases was equal (Table 4). The majority of the corticosteroid users were in the group of refractory MC (*p* = 0.015), with no difference regarding other drug treatments. The symptoms constipation and bloating and flatulence were most pronounced in the group of one episode of MC (Table 4). Ten patients with one episode of MC suffered from constipation at inclusion (4 patients with LC), in combination with diarrhea in 4 cases. Only three patients in the refractory group had constipation, in combination with diarrhea in two cases.

**Table 3:** Basal characteristics depending on one episode of MC or refractory microscopic colitis

	**One episode of MC** ***N* = 70**	**Refractory MC** ***N* = 88**	***P*-value**
**Age** (years)	63.8 (59.5–67.3)	62.5 (56.5–67.6)	0.481
**BMI** (kg/m^2^) *Missing value*	25.4 (24.0–29.2) 32	24.1 (21.7–27.5) 38	0.063
**CC/LC**	36 (51.4)/34 (48.6)	56 (63.6)/32 (36.4)	0.145
**IBS**	39 (55.7)	48 (54.5)	1.00
**Disease duration** (years) *Missing value*	6.5 (3.0–12.0) 8	10.0 (4.0–16.0) 7	0.200
**University education** *Missing value*	21 (30.0) 2	33 (37.5) 2	0.623
**Occupation**			0.203
Employed	27 (38.6)	43 (48.9)	
Retired	37 (52.9)	42 (47.7)	
Others	6 (8.6)	3 (3.4)	
**Married/living together** *Missing value*	40 (57.1) 1	49 (55.7)	0.871
**Smoking**			0.007
Never smoking	14 (20.0)	27 (30.7)	
Past smoking	38 (54.3)	26 (29.5)	
Present smoking	18 (25.7)	35 (39.8)	
**Snuff users** *Missing value*	4 (5.7) 2	0 4	0.038
**Nicotine chewing gum** *Missing value*	4 (5.7) 1	10 (11.4) 2	0.266
**The number of days drinking/month** *Missing value*	4 (2–10) 18	5 (2–10) 23	
**Number of standard glasses each day of drinking**			0.153
Never drinking	6 (8.6)	9 (10.2)	
1	6 (8.6)	11 (19.6)	
2	19 (27.1)	31 (35.2)	
3	9 (12.9)	3 (3.4)	
4	3 (4.3)	2 (2.3)	
≥ 5	2 (2.8)		
*Missing value*	25	32	

*BMI* body mass index, *CC* collagenous colitis, *IBS* irritable bowel syndrome, *LC* lymphocytic colitis, *MC* microscopic colitis. Values are given as number and percentage and median and interquartile range. Mann–Whitney U test and Fisher´s exact test. *P* ≤ 0.05 was considered statistically significant

**Table 4:** Comorbidity and symptoms depending on one episode of MC or refractory microscopic colitis

	**One episode of MC** ***N* = 70**	**Refractory MC** ***N* = 88**	***P*-value**
**Comorbidity**			
Hypertension *Missing value*	24 (34.3) 7	30 (34.1) 9	1.000
Rheumatoid arthritis *Missing value*	14 (20.0) 8	19 (21.6) 14	0.473
Asthma and/or bronchitis *Missing value*	13 (18.6) 9	12 (13.6) 14	0.726
Diabetes *Missing value*	4 (5.7) 10	7 (8.0) 14	0.836
Celiac disease *Missing value*	9 (12.9) 14	2 (2.3) 16	0.010
Gastric ulcer *Missing value*	10 (14.3) 8	13 (14.8) 14	0.518
Thyroid disease *Missing value*	16 (22.9) 10	12 (13.6) 15	0.200
Cancer *Missing value*	3 (4.3) 11	10 (11.4) 15	0.190
**Drug treatment**			
Corticosteroids	14 (20.0)	34 (38.6)	0.015
Proton pump inhibitors	14 (20.0)	27 (30.7)	0.147
Selective serotonin reuptake inhibitors	10 (14.3)	20 (22.7)	0.222
Statins	13 (18.6)	15 (17.0)	0.836
Thyroid hormones	14 (20.0)	12 (13.6)	0.382
**Gastrointestinal symptoms**			
Abdominal pain 5 (1–15)	45 (13–54)	30 (12–53)	0.195
Diarrhea 3 (0–10)	48 (19–69)	46 (20–77)	0.826
Constipation 9 (1–22)	13 (5–32)	5 (2–14)	< 0.001
Bloating and flatulence 14 (1–29)	58 (31–77)	48 (18–70)	0.039
Vomiting and nausea 2 (0–3)	10 (3–44)	5 (1–23)	0.056
Intestinal influence on daily life 2 (0–18)	46 (18–82)	42 (18–76)	0.564
Psychological well-being 4 (0–16)	26 (9–50)	24 (11–54)	0.905

Comorbidity reflects whether the patients ever have been treated for any of these diseases. Missing values mean that the participant has not answered the question whether they have had the disease or not in the questionnaire. Gastrointestinal symptoms were assessed by the Visual Analog Scale for Irritable Bowel Syndrome, 0–100 mm, where 0 represents no symptoms and 100 maximal symptoms [20]. Reference values for VAS-IBS from healthy women are shown within brackets [21]. Values are given as number and percentages or median and interquartile ranges. Mann–Whitney U test or Fisher´s exact test. *P* ≤ 0.05 was considered statistically significant

After screening of anti-tTG in all patients according to clinical routine analysis, only one patient expressed positive antibody titers (> 7 kU/L), and she had had one episode of LC and already diagnosed with celiac disease. A second in-house screening among the refractory MC cases, showed that all were in the normal ranges, and no one exhibited positive antibodies (5.9 (4.4–7.9) EU/ml).

### Smoking and snuff habits

Only 41 participants had never been smoking, whereas 64 participants were former smokers, and 52 participants were present smokers. When the patients were divided into smoking habits, the group of past smoking had the lowest prevalence of refractory MC (40.6%; *p* = 0.007). IBS was most common among present smokers (69.8%; *p* = 0.030). Since the group of present smokers was younger, they were also to a higher degree employed instead of retired and were more often married or living together. On the opposite, the disease duration was longer (Table 5). The prevalence of celiac disease was highest in the group of past smoking. Present smokers had more bloating and flatulence, intestinal influence of symptoms on daily life, and impaired psychological well-being than non- and past smokers (Table 6).

**Table 5:** Basal characteristics depending on smoking habits

	**Never smoking** ***N* = 41**	**Past smoking** ***N* = 64**	**Present smoking** ***N* = 53**	***P*-value**
**Age** (years)	64.9 (57.1–69.8)	65.3 (60.8–69.3)	60.0 (54.2–64.0)	< 0.001
**BMI** (kg/m^2^) *Missing value*	25.8 (24.1–29.9)	24.8 (23.0–28.7)	24.1 (21.7–26.6)	0.164
**Refractory MC**	27 (65.9)	26 (40.6)	35 (66.0)	0.007
**CC/LC**	23 (56.1)/18 (43.9)	36 (56.3)/28 (43.8)	33 (62.3)/20 (37.7)	0.788
**IBS**	19 (46.3)	31 (48.4)	37 (69.8)	0.030
**Disease duration** (years) *Missing value*	7.0 (3.0–12.0) 5	6 (3.0–11.0) 4	10.0 (6.0–21.0) 6	0.021
**University education** *Missing value*	16 (39.0) 1	17 (26.6) 1	21 (3.8) 2	0.397
**Occupation**				0.035
Employed	16 (39.0)	25 (39.1)	29 (54.7)	
Retired	24 (58.5)	37 (57.8)	18 (34.0)	
Others	1 (2.4)	2 (3.1)	6 (11.3)	
**Married/living together** *Missing value*	28 (68.3)	39 (60.9)	22 (41.5) 1	0.031
**Snuff user** *Missing value*	1 (2.4) 3	3 (4.7) 1	0	0.363
**Nicotine chewing gum** *Missing value*	0 2	4 (6.3) 1	10 (18.9)	0.005
**The number of days drinking/month** *Missing value*	4 (1–8) 11	6 (3–10) 14	4 (0–9) 16	0.088
**Number of standard glasses each day of drinking**				0.107
Never drinking	3 (7.3)	3 (4.7)	9 (17.0)	
1	7 (17.1)	5 (7.8)	5 (9.4)	
2	13 (31.7)	25 (39.1)	12 (22.6)	
3	1 (2.4)	7 (10.9)	4 (7.5)	
4	1 (2.4)	3 (4.7)	1 (1.9)	
≥ 5		1 (1.6)	1 (1.9)	
*Missing value*	16	20	21	

*BMI* body mass index, *CC* collagenous colitis, *IBS* irritable bowel syndrome, *LC* lymphocytic colitis, *MC* microscopic colitis. Values are given as number and percentage and median and interquartile range. Kruskal–Wallis test and Fisher´s exact test. *P* ≤ 0.05 was considered statistically significant

**Table 6:** Comorbidity and symptoms depending on smoking habits

	**Never smoking** ***N* = 41**	**Past smoking** ***N* = 64**	**Present smoking** ***N* = 53**	***P*-value**
**Comorbidity**				
Hypertension *Missing value*	11 (26.8) 5	26 (40.6) 7	17 (32.1) 4	0.541
Rheumatoid arthritis *Missing value*	9 (22.0) 5	13 (20.3) 12	11 (20.8) 5	0.760
Asthma and/or bronchitis *Missing value*	6 (14.6) 6	9 (14.1) 13	10 (18.9) 4	0.492
Diabetes *Missing value*	5 (12.2) 5	4 (6.3) 14	2 (3.8) 5	0.299
Celiac disease *Missing value*	3 (7.3) 6	8 (12.5) 15	0 9	0.009
Gastric ulcer *Missing value*	7 (17.1) 1	7 (10.9) 11	9 (17.0) 4	0.607
Thyroid disease *Missing value*	6 (14.6) 5	14 (21.9) 11	8 (15.1) 9	0.529
Cancer *Missing value*	2 (4.9) 6	4 (6.3) 14	7 (13.2) 6	0.396
**Drug treatment**				
Corticosteroids	11 (26.8)	19 (29.7)	18 (34.0)	0.759
Proton pump inhibitors	14 (34.1)	15 (23.4)	12 (22.6)	0.392
Selective serotonin reuptake inhibitors	9 (22.0)	9 (14.1)	12 (22.6)	0.410
Statins	6 (14.6)	15 (23.4)	7 (13.2)	0.349
Thyroid hormones	6 (14.6)	13 (20.3)	7 (13.2)	0.533
**Gastrointestinal symptoms**				
Abdominal pain 5 (1–15)	31 (13–50)	31 (6–54)	41 (17–59)	0.154
Diarrhea 3 (0–10)	46 (16–62)	40 (14–73)	54 (28–80)	0.133
Constipation 9 (1–22)	8 (2–20)	8 (2–25)	8 (3–22)	0.871
Bloating and flatulence 14 (1–29)	49 (18–60)	47 (22–75)	63 (28–80)	0.044
Vomiting and nausea 2 (0–3)	5 (0–19)	5 (2–23)	14 (3–47)	0.065
Intestinal influence on daily life 2 (0–18)	36 (16–72)	36 (12–71)	64 (28–89)	0.012
Psychological well-being 4 (0–16)	22 (6–34)	22 (10–48)	40 (12–67)	0.025

Comorbidity reflects whether the patients ever have been treated for any of these diseases. Missing values mean that the participant has not answered the question whether they have had the disease or not in the questionnaire. Gastrointestinal symptoms were assessed by the Visual Analog Scale for Irritable Bowel Syndrome, 0–100 mm, where 0 represents no symptoms and 100 maximal symptoms [20]. Reference values for VAS-IBS from healthy women are shown within brackets [21]. Values are given as number and percentages or median and interquartile ranges. Kruskal–Wallis test or Fisher´s exact test. *P* ≤ 0.05 was considered statistically significant

Snuff using was unusual and only occurred in 2.5% of the patients (Table 1). Snuff use did not differ related to histopathological findings (*p* = 1.000) or smoking habits (*p* = 0.363) but was most prevalent in patients with one episode of MC (*p* = 0.038) (Table 1, 3, and 5). Nicotine chewing gum was used by 8.9% and was most often used by present smokers (*p* = 0.005) (Table 5).

### Associations with clinical expression

About half of the patients with celiac disease had their symptom debut and/or diagnoses of celiac disease and MC at the same period (Table 7). In three of the cases, the celiac disease diagnosis was set first, in two cases, the diagnoses were set simultaneously, and in one case, the MC diagnosis was set first. In the other five cases, the diagnosis of MC was set after celiac disease (Table 7). The two diagnoses were set independent of each other.

**Table 7:** The age at baseline for inclusion, symptom debut and diagnoses

Cases	Age at study inclusion (year)	Age at celiac disease diagnosis (year)	Age at symptoms of MC (year)	Age at MC diagnosis (year)
1	71	48	65	66
2	61	36	36	53
3	28	21	20	25
4	67	51		58
5	68	60	59	60
6	63	61	61	61
7	72	67		65
8	66	50	64	65
9	71	40	50	71
10	57	20	56	57
11	72	30	30	32

The age in years when the diagnoses of celiac disease was set and when the symptoms of microscopic colitis and its diagnosis was set

Smoking cessation preceded the debut of CC in 68.6% of cases and LC in 85.2% of cases, and in 75.0% of one episode of MC and in 76.9% of refractory MC.

When performing logistic regression with adjustment for smoking habits, celiac disease was associated with LC (OR: 4.222; 95% CI: 1.020–17.469; *p* = 0.047) and tended to be inversely associated with refractory MC (OR: 0.210; 95% CI: 0.042–1.506; *p* = 0.058). Smoking habits were not associated with histopathological findings or clinical course (data not shown).

## Discussion

The main finding in the present study was the consolidation of previous literature on the association between LC and celiac disease [9, 14, 15]. Furthermore, celiac disease seems to be more prevalent in patients with one episode of MC and past smokers.

MC is considered a chronic disease and is often treated for long time with corticosteroids. Nevertheless, we have previously described that MC may occur and be documented as one episode, although MC is classified as a chronic disease [27]. Past smoking was associated with one episode of MC, and current smoking was associated with refractory MC, when all cases with celiac disease were excluded [28]. The current findings that almost half of the patients only had had one single episode of MC, based on the patient reports and medical records, is in accordance with a large prospective European cohort study which demonstrated a chronic course in only 49% of cases, independently of subtype [29]. In contrast, another study described that 63% of LC only have a single episode of the disease, compared to 30% exhibiting a chronic recurrent course [15]. One episode of MC was much more uncommon in the CC group (2%) [15]. The follow-up time from diagnosis was several years both in the former and present studies, which strengthens the findings that the disease may present only as a single episode [15, 27, 29]. There were no clinical differences between CC and LC, except that LC was associated with celiac disease and CC with treatment of corticosteroids. Therefore, the most important is possibly not to stress the histopathological findings, but to follow the disease course.

The hypothesis that MC may be the colonic reaction to gluten in celiac disease [9, 14, 15] was supported since 9 out of 11 patients with celiac disease only had had one episode of MC, which in half of the cases had debuted simultaneously. To confirm this hypothesis, a prospective study must be performed with duodenal and colonic biopsies before and after exclusion of gluten. However, we suggest greater caution before the clinical diagnosis of chronic MC is set [27]. Relapses of the disease must be observed during the follow-up before the definitive diagnosis is set, in similarity to other IBD diseases [22, 23, 30, 31], and secondary forms of mucosal lymphocytic infiltrates must be excluded, e.g., celiac disease, infections, and drugs [14]. Duodenal intraepithelial lymphocytosis is a type of inflammation associated with MC [7], although this is a frequent finding after a transient gastrointestinal infection and in gluten sensitivity [14, 32]. Furthermore, the subtype of MC may shift over time, from one entity to the other, and from MC to other IBDs [33, 34].

The present association between MC and celiac disease is well described. An American case–control study found a prevalence of celiac disease in 4.4% of MC patients, and MC was diagnosed in 10.0% of patients with celiac disease [7]. In a histopathological study of celiac disease, 19.0% of cases also had LC at diagnosis [35], and as many as 30% of patients with celiac disease had MC according to a review of celiac disease [36]. The association was strongest between LC and celiac disease in both American and Swedish cohort studies [7, 9], in similarity with our MC cohort, but in opposite to a Danish cohort [8]. The prevalence of celiac disease in a Scandinavian CC cohort was 12.9% [5], in comparison to the prevalence of about 2% in the Swedish general population [37]. The differences of prevalence between studies may depend on assorted designs. The Scandinavian study [5] was performed in a University Hospital setting, which means an accumulation of selected cases, whereas the American study was performed in all kinds of health care centers [7]. In a systematic review with meta-analysis, the prevalence of MC in refractory celiac disease was 4.5%, and the prevalence of celiac disease in refractory MC was 6.7% [38]. Comparable results were found in a second review [39]. The prevalence of celiac disease was eight times higher in refractory MC compared to controls [38], which underlines the importance of excluding gluten intolerance in MC patients who do not respond to conventional therapy. Also, patients with celiac disease and persistent symptoms must be examined to exclude MC [40]. Analysis of anti-tTG in the current study excluded celiac disease in the cases with refractory MC. The question must be raised whether MC and celiac disease are two different entities when occurring in the same patient, or whether the MC in some circumstances should be considered as a component of the celiac disease with lymphocytic invasion of the mucosa [14].

The celiac disease diagnosis was set a few years prior to the CC diagnosis in most cases [5]. In another Scandinavian study of LC, 8 of 17 cases with celiac disease were diagnosed before the LC diagnosis, whereas the diagnoses were set at the same time in 9 of 17 cases [15]. In an American study, 64% were diagnosed with celiac disease before MC, while 25% were diagnosed with both disease in the same month, with 75% diagnosed as LC [41]. Those with LC had more severe villus atrophy than those with CC [41]. The risk of MC diagnosis was far higher during the first year after the celiac disease diagnosis than after 10 years in a nationwide Swedish cohort [9]. When MC and celiac disease are diagnosed in the same patient, the age is in general 10 years lower compared with the age in patients with pure MC [9]. Treatment with gluten withdrawal may heal both the villus atrophy and the colonic alterations [15]. This raises the hypothesis whether gluten-free diet would be a treatment option in MC, especially in LC. At least, diagnosis of MC should always be accompanied with exclusion of celiac disease with antibody screening and/or gastroscopy and duodenal biopsy [3]. Diagnosis of MC some years after celiac disease may depend on poor compliance to the diet. A higher risk of MC development has been related to an untreated celiac disease or suboptimal adherence to gluten-free diet [9]. The dietary compliance was not examined in the current cohort. This is an important issue, since patients diagnosed with celiac disease through screening programs in adults is not associated with worse adherence to the gluten-free diet and might protect from complications such as MC or metabolic bone disease [42].

Constipation and bloating are associated symptoms. The higher degree of these symptoms in MC with only one episode compared to refractory MC, suggest that MC with one episode is another disease with other etiologies than refractory MC. Constipation in MC may depend on varying factors such as comorbidity, drug treatments, use of antidiarrheals, physical inactivity, or difficulties to empty the bowel due to pelvic floor dysfunction. Since IBS-like symptoms are that common in MC, it may be difficult to evaluate disease activity and correct diagnosis, without mucosal biopsies [19, 27].

Several patients were on continuous treatment with corticosteroids, although they only had had one relapse of the disease and normal biopsies in subsequent examinations. This may depend on that the physician believes that the treatment may prevent relapses. The high prevalence of MC with one episode stresses the need for restrictions in the treatment. Corticosteroid use may lead to dependence and misuse [43], leading to a challenge to quit corticosteroid treatment. Chronic use of corticosteroid is related to side effects, e.g., increased rates of infection, adrenal suppression, osteoporosis, diabetes, cardiovascular events, mood disorders, and all-cause mortality [43].

It has previously been described that not only the disease of MC is more common in smokers; the disease also debuts earlier in life in smokers compared with non-smokers [44, 45], reflected by the longer disease duration in smokers in the current study. The higher degree of working in present smokers probably reflects the younger age in that group. IBS-like symptoms and impaired psychological well-being were most frequently found in present smokers. This is in accordance with findings in systematic reviews and in population-based studies, which have described IBS to be associated with smoking [46, 47]. In the former study describing association between former smoking and MC with one episode in comparison to current smoking and refractory MC, all cases with celiac disease were excluded not to interact with the MC diagnosis when studying smoking and alcohol habits regarding MC [28]. When including celiac disease in the present study, we found high prevalence of past smokers in the patients with celiac disease and MC with one episode. Smoking has no association with celiac disease [48]. Accordingly, past smoking was the most common smoking habit in the current subgroup of MC patients with one episode of the disease and in those with celiac disease. As described previously in US women [49], current smoking appeared to be stronger associated with CC than with LC, also found in a meta-analysis [50]. Even if the subject has ceased smoking, the effect of chronic smoking may have affected the mucosa and promoted disease development [49, 51]. Smoking leads to inhalation of several different compounds, with varying effect on cellular and tissue functions of the intestinal mucosa such as the mucosal barrier, gut microbiota, immune system, and microvasculature [52]. Further, it may be difficult to remember exactly when the symptoms debuted, especially if the symptoms are starting gradually. Snuff and nicotine chewing gums were of no major importance, since the uses were rather uncommon.

To use the histopathological diagnosis as a clinical diagnosis, without considering secondary causes or one episode of LC [14], will be misleading for the prevalence of MC. Several associations may be found between MC and concomitant diseases and drug only due to the higher age of the patient group [9, 53]. The present, liberal attitude to diagnosis of MC after one single episode, weakens the trustworthiness of research and may obscure the true disease through inclusion of several heterogenous patient cohorts. The tendency of over diagnosing symptoms must be questioned, since there is a danger of aggravating people’s perception of their symptoms and over prescription of drugs, thus, increasing the cost to the society of health care. Further, to receive a diagnosis of a chronic life-long disease may be a stigma for the individual and weaken the possibilities to get life insurances. Debut of a new disease, e.g., malignancy, may be neglected since the symptoms may be referred to MC and new examinations leading to a correct diagnosis delayed.

The novelty of the current study is the classification into one episode of MC or refractory MC, considering smoking as a confounder, when calculating the association between celiac disease and MC. There are several limitations in the present study. First, there is no adjustment for drug treatments and no control material of healthy individuals. However, a control population and considerations of drug treatments have been used in other studies [1, 3, 28]. Only women were included, since the prevalence of MC in men was very uncommon in the area, and thus, no evaluations could be performed in male patients. The retrospectively collected data is another limitation. To verify the hypothesis that MC may be the colonic reaction to gluten in celiac disease, a prospective study of consecutively diagnosed patients with celiac disease must be performed with careful biopsy sampling from both the small and large intestine at the same time points, and sample collection both before and after introduction of gluten-free diets.

In conclusion, a great deal of patients with MC has a single episode of the disease and should not be assigned to a chronic MC disease, as has previously been described in a large prospective study [18]. The first line of treatment of MC should be exclusion of celiac disease as previously proposed in European guidelines [3]. The close association between MC and celiac disease must be recognized in cases with symptoms refractory to conventional treatment of either disease. The accurate nomenclature of diagnoses when both LC and celiac disease are diagnosed simultaneously should be further discussed, to define whether it is the same or two different entities, especially when there is only one episode of LC.

## Data Availability

Due to ethical rule, the data set is not provided, but can be provided by the corresponding author upon request.

## Abbreviations

CC: Collagenous colitis
IBD: Inflammatory bowel disease
IBS: Irritable bowel syndrome
LC: Lymphocytic colitis
MC: Microscopic colitis
VAS-IBS: Visual analog scale for irritable bowel syndrome
